# A VCG-Based Multiepitope *Chlamydia* Vaccine Incorporating the Cholera Toxin A1 Subunit (MECA) Confers Protective Immunity Against Transcervical Challenge

**DOI:** 10.3390/biomedicines13020288

**Published:** 2025-01-24

**Authors:** Fnu Medhavi, Tayhlor Tanner, Shakyra Richardson, Stephanie Lundy, Yusuf Omosun, Francis O. Eko

**Affiliations:** Department of Microbiology, Biochemistry and Immunology, Morehouse School of Medicine, Atlanta, GA 30310, USA; fmedhavi@msm.edu (F.M.); yomosun@msm.edu (Y.O.)

**Keywords:** *Chlamydia trachomatis*, rVCG-MECA, vaccine delivery, route, protective immunity

## Abstract

**Background/Objectives**: We generated a novel recombinant *Vibrio cholerae* ghost (rVCG)-based subunit vaccine incorporating the A1 subunit of cholera toxin (CTA1) and a multiepitope *Chlamydia trachomatis* (CT) antigen (MECA) derived from five chlamydial outer membrane proteins (rVCG-MECA). The ability of this vaccine to protect against a CT transcervical challenge was evaluated. **Methods**: Female C57BL/6J mice were immunized thrice at two-week intervals with rVCG-MECA or rVCG-gD2 (antigen control) via the intramuscular (IM) or intranasal (IN) route. PBS-immunized mice or mice immunized with live CT served as negative and positive controls, respectively. **Results**: Vaccine delivery stimulated robust humoral and cell-mediated immune effectors, characterized by local mucosal and systemic CT-specific IgG, IgG2c, and IgA antibody and IFN-γ (Th1 cytokine) responses. The elicited mucosal and systemic IgG2c and IgA antibody responses persisted for 16 weeks post-immunization. Immunization with rVCG-MECA afforded protection comparable to that provided by IN immunization with live CT EBs without any side effects, irrespective of route of vaccine delivery. **Conclusions**: The results underline the potential of a multiepitope vaccine as a promising resource for protecting against CT genital infection and the potential of CTA1 on the VCG platform as a mucosal and systemic adjuvant for developing CT vaccines.

## 1. Introduction

*Chlamydia trachomatis* (CT), an obligate intracellular, Gram-negative-like bacterium, is the most prevalent sexually transmitted infection (STI) worldwide. In 2022, the CDC reported over 1.6 million cases in the United States alone, highlighting its persistent prevalence [1]. One of the major challenges in controlling the spread of CT is its asymptomatic nature, with more than 70% of infections going undiagnosed and untreated [2,3]. Untreated CT infection in women can lead to severe complications, including pelvic inflammatory disease (PID), chronic pelvic pain, ectopic pregnancy, and tubal factor infertility, conditions that significantly affect reproductive health [4,5]. It is estimated that PID develops in 10–25% of infected women, with problems such as chronic pelvic pain, tubal factor infertility, and ectopic pregnancies occurring in 10–30% of cases [6]. Additionally, CT enhances the risk of HIV-1 acquisition and transmission [7] and human papilloma virus-associated cervical dysplasia [8]. Common symptoms in men include inflammation of the urethra, epididymis, and testicles, although long-term sequelae are less frequently observed [9].

Given the substantial public health burden and the severe long-term health impacts, including infertility and an increased risk of certain cancers, the development of prevention strategies is crucial [10]. While antibiotic treatment is effective, the infection is allowed to spread widely due to the lack of overt symptoms, underscoring the need for additional public health interventions. Vaccine development is a vital strategy to combat the ongoing challenge of CT infection and its associated complications [11,12,13]. Despite its prevalence and significant burden on public health, there is currently no licensed *Chlamydia* vaccine. *Chlamydia* vaccine development has faced several challenges, including the bacterium’s unique intracellular lifecycle and its ability to evade the host immune response [14,15].

Historically, following the discovery that inactivated *Chlamydia* vaccines induced pathology in recipients [16,17], vaccine development efforts shifted to the identification of immunogenic antigens capable of providing long-lasting protection [15]. Development of subunit vaccines imposes the requirement for an effective delivery method capable of inducing local mucosal and systemic immune responses. Recent advances in vaccine technology have introduced novel vaccine delivery strategies, including the *Vibrio cholerae* ghost (VCG) vaccine delivery platform, for targeting specific *Chlamydia* antigens to the immune system to elicit protective immune responses [18]. VCG, which are empty bacterial cell envelopes, are derived from *V. cholerae* by a genetic inactivation procedure that causes the release of cellular cytoplasmic contents, including cholera toxin while preserving the immunogenic surface components [19]. The VCG platform has been shown to boost the protective immunity afforded by delivered vaccine antigens without the use of adjuvants [20,21]. Another critical factor influencing vaccine efficacy is the route of administration, as different routes can lead to varying immune outcomes. For pathogens like CT, which primarily infect mucosal surfaces, both systemic and mucosal immunity are crucial for effective protection [22]. Systemic vaccination is traditionally associated with strong systemic immune responses, characterized by high levels of circulating antibodies and activated T cells, while mucosal vaccination is expected to induce mucosal immunity, which is particularly important for preventing infections at mucosal sites [21]. The enzymatically active CTA1-subunit of cholera toxin (Ctx) has been shown to be an effective mucosal and systemic adjuvant, stimulating strong cellular and humoral immune responses [23,24,25] and is non-toxic and safe following delivery by the intranasal route [26].

Different inbred mouse strains have been employed in various research fields. The most commonly used mouse strains in *Chlamydia* vaccine research include BALB/c, C57BL/6J, and C3H/HeJ. A previous report indicates that the macrophage function of BALB/c is severely impaired compared to C57BL/6J mice [27] and BALB/c mice generally produce comparably higher levels of IgG antibody responses after antigen stimulation, making them suitable for use as a mouse model for investigating humoral immune responses. C57BL/6J mice are widely used in immunological research due to their well-characterized genome [28] and strong Th1-type immune responses [29], which is essential for combating intracellular pathogens like CT. Protection against CT genital infection is dependent primary on IFN-γ-secreting CD4+ Th1 cells [30]. While their lower antibody responses compared to other strains like BALB/c and A/J mice are a limitation in certain studies, their relevance to Th1-skewed immunity [29] makes them ideal in CT vaccine evaluation studies.

In this study, we compared the efficacy afforded by a VCG-based multiepitope CT antigen derived from CT outer membrane proteins fused to CTA1 (rVCG-MECA) following intramuscular (IM) systemic and intranasal (IN) mucosal administration. These proteins, polymorphic membrane protein D (PmpD), PmpC, PmpG, PorB, and outer membrane complex B (OmcB), play critical roles in chlamydial pathogenesis and are prime targets for vaccine development [13,18]. The study revealed that vaccine delivery induced robust protective immunity comparable to that provided by IN immunization with live CT EBs, indicating the ability of VCG-based epitope vaccines to induce protective immunity following both systemic and mucosal administration.

## 2. Materials and Methods

### 2.1. Ethics Statements

This research was performed in strict adherence to the guidelines outlined in the National Institutes of Health Guide for the Care and Use of Laboratory Animals. The study was approved by the Institutional Animal Care and Use Committee (IACUC) of the Morehouse School of Medicine (Assurance number A3381-01) (IACUC Approval # 19-09).

### 2.2. Chlamydia Stocks, Antigens and Mice

Stock preparations of *C. trachomatis* serovar D used in this study were previously titrated on Mccoy cell monolayers, followed by purification of elementary bodies (EBs) by centrifugation over renografin gradients (Virusys Corporation, Randallstown, MD, USA), and stored at −80 °C. CT antigen (coating and restimulation antigen) was prepared by UV-inactivation of EBs for 3 h and stored at 80 °C until used.

The study utilized six-week-old inbred female C57BL6/J mice (stock number 000664) obtained from The Jackson Laboratory (Bar Harbor, ME, USA). The mice underwent a three-day period of acclimatization at the MSM Center for Laboratory Animal Resources facility prior to experimentation. The mice were given food and water ad libitum. All immunizations and challenges were performed under isoflurane (4%) anesthesia and the mice were monitored for any signs of adverse effects, such as ruffled hair, pilomotor response (hair raising), remaining immobile, guarding the injection site, and dyspnea. No signs of adverse effects were observed and so none were reported. At the end of the experiment, the mice were euthanized by CO_2_ asphyxiation and cervical dislocation as recommended by the Panel on Euthanasia of the American Veterinary Medical Association.

### 2.3. Construction of the Vaccine Vector, pCT-MECA, and Expression of MECA by Immunoblotting Analysis

Immunogenic epitopes from five outer membrane proteins, PmpD, PmpC, PmpG, PorB, and OmcB, previously shown to be immunogenic [31,32,33,34], were predicted. The selected epitopes were fused together using GPGPG linkers together with the A1 subunit of the cholera toxin (CTA1) at the N-terminal region and termed the multiepitope CT antigen (MECA). For the construction of the vaccine vector, the 660 amino acids of the MECA were reverse-translated into a DNA coding sequence. The sequence was codon optimized to enhance the expression in *V. cholerae* and synthesized to contain Xho I and Kpn I restriction sites at the N- and C-terminal ends (GenScript, Piscataway, NJ, USA).

**Figure 1:**
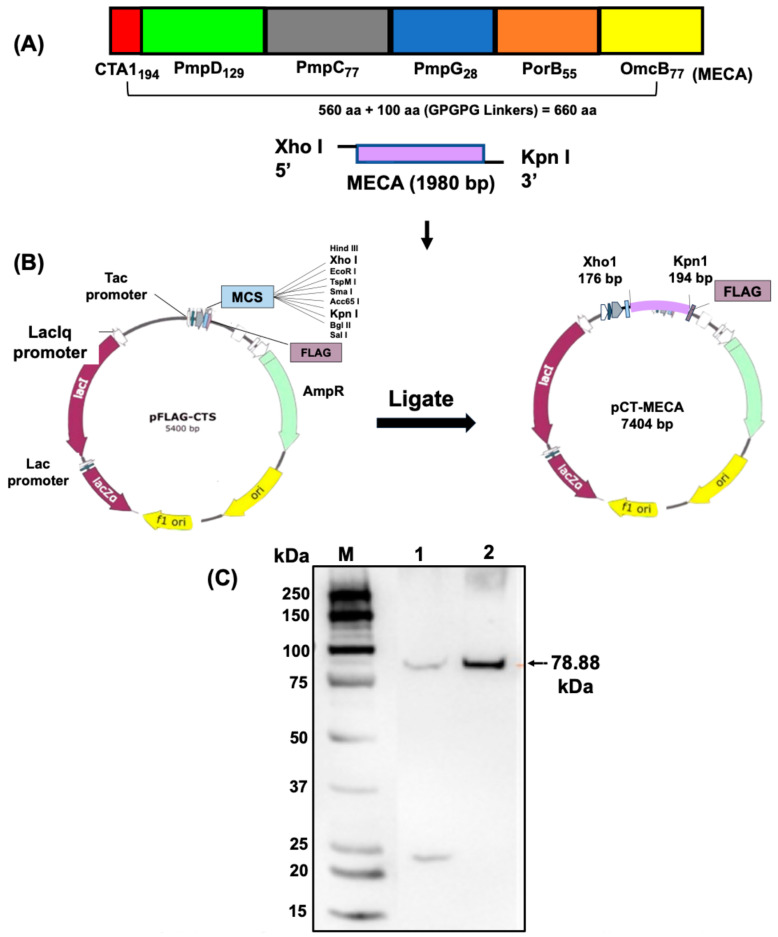
Design and construction of the vaccine vector, pCT-MECA, and expression of rMECA. (**A**) Twenty (20) immunogenic T and B cell epitopes from five CT outer membrane proteins were selected and fused with CTA1 using linkers. (**B**) The 1980 bp synthesized coding sequence was inserted into the periplasmic targeting expression vector, pFLAG-CTS, in frame with the Flag Tag sequence to generate plasmid pCT-MECA. (**C**) Following transformation of *V. cholerae* V912 harboring the pDKLO1 lysis plasmid with plasmid pCT-MECA and production of rVCG-MECA, the expression of rMECA was confirmed by Western immunoblotting analysis of lyophilized rVCG-MECA samples using anti-Flag monoclonal antibodies. Lane 1—uninduced pCT-MECA control. Lane 2—rMECA 4 h post IPTG induction. MW—Molecular weight marker in kilodaltons (kDa).

The synthesized gene (1980 bp) was then inserted into the periplasmic targeting expression vector, pFLAG-CTS, using Xho I and Kpn I restriction sites incorporated into the gene (GenScript, Piscataway, NJ, USA). The resultant plasmid was designated pCT-MECA (Figure 1) and used to transform by electroporation *V. cholerae* O139 strain V912 harboring the pDKLO1 plasmid, which expresses the lysis protein E. Cells grown in brain heart infusion broth at 37 °C were induced for protein expression by addition of isopropyl β-D-thiogalactopyranoside (IPTG) (Roche Diagnostics, Indianapolis, IN, USA) followed by gene *E*-mediated lysis as previously described [18]. Following expression, recombinant MECA was released and localized in the periplasmic space of *V. cholerae*, where it was sequestered upon lysis. The lysed cells were harvested, washed, freeze-dried, and MECA protein expression was confirmed by western immunoblotting analysis using an anti-FLAG monoclonal antibody (Invitrogen, Waltham, MA, USA) and imaged using the ChemiDoc MP Imagining System version 1.4.3.

### 2.4. Production of rVCG-MECA Vaccine

*V. cholerae* strain V912 cells harboring pCT-MECA and the lysis plasmid pDKLO1 were cultured in Brain Heart Infusion (BHI) broth at 37 °C to mid-log phase and rVCG-MECA was produced by genetic inactivation of the cells involving the addition of 3-methyl benzoate to a growing culture, as previously described [18]. The cells were harvested at the end of lysis, washed with a low ionic buffer, and lyophilized. The lyophilized rVCG-MECA were stored in cryovials at room temperature until used.

### 2.5. Immunization and Challenge of Immunized Mice

**Figure 2:**
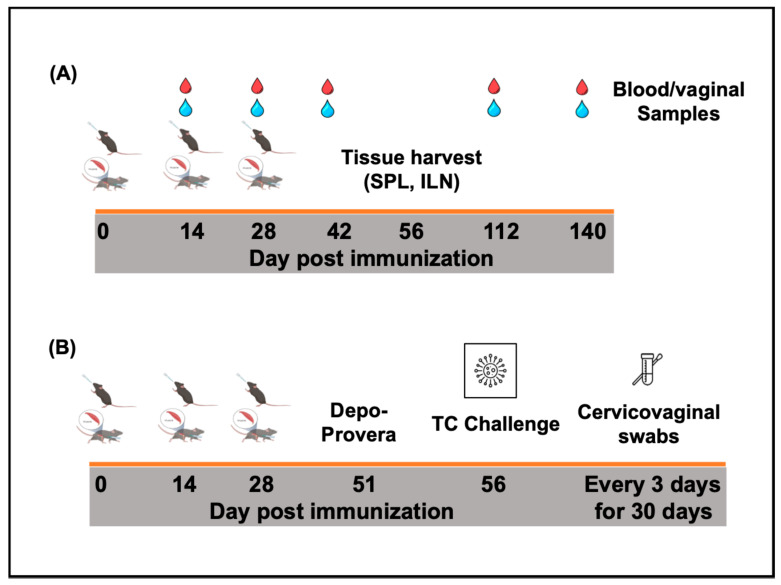
Schematic diagram of the experimental protocol outlining the immunization, sample collection (**A**), and challenge (**B**) schedules.

For immunogenicity studies (Figure 2A), groups of female C57BL/6 mice (16/group) were immunized either intramuscularly, IM (50 mL), or intranasally, IN (20 mL), and boosted two weeks apart with PBS containing 1.5 mg of lyophilized rVCG-MECA or rVCG-gD2 (VCG expressing the glycoprotein D2 from HSV-2, a chlamydial irrelevant antigen) (vaccine antigen control). PBS-immunized mice or mice immunized IN with 1 × 10^6^ IFU of live *C. trachomatis* (CT) EBs served as negative and positive controls, respectively. Blood and vaginal washes were collected on days 14, 28, and 42 as well as on days 112 and 140 (i.e., 12 and 16 weeks after the last immunization) to assess long-term antibody responses. Four weeks after the last immunization (day 56), the mice designated for cell-mediated immune response studies (8/group) were sacrificed and the immune T cells were purified from the harvested spleens (SPL) and iliac lymph nodes (ILN) of the immunized mice using the gentleMACS Dissociator.

In a separate experiment (Figure 2B), groups of mice (8/group) were immunized as described above. Three weeks after the last immunization, the mice designated for challenge studies (8/group) were treated subcutaneously with Depo-Provera (2.5 mg/mouse; UpJohn Co., Kalamazoo, MI, USA) to synchronize the estrous cycle and enhance susceptibility to infection and challenged transcervically one week later (four weeks after the last immunization) with live *C. trachomatis* serovar D (1 × 10^6^ IFU/mouse). To monitor bacterial load and time to clearance of infection, cervicovaginal swabs were collected every three days for 30 days. The mice were observed daily to assess their health status, and infection levels were determined by quantifying chlamydial inclusion-forming units (IFUs) from cervicovaginal swabs using indirect immunofluorescence [35]. The mean number of IFUs was then calculated at each time point.

### 2.6. Determination of Antigen-Specific Humoral Immune Responses

CT-specific antibodies (IgG, IgG2c, and IgA) in serum and vaginal wash samples collected at designated time points after immunization and IgG1 from serum obtained 2 weeks after the last immunization were measured using a standard ELISA technique described previously [36]. Briefly, Maxisorb 96-well plates (Costar) were coated with CT antigen (UV inactivated CT Serovar D EBs) at a concentration of 10 mg protein/mL in sodium carbonate. To create a standard calibration curve, wells were coated in triplicate with varying concentrations of IgA or IgG2c standards (ranging from 0 to 1000 ng/mL). The plates were then blocked with a solution of 1% bovine serum albumin and 5% goat serum in PBST. This was followed by the addition of serum samples (diluted 1:20) and vaginal wash samples (diluted 1:100), with each sample being incubated in the wells. After this, 50 mL of horseradish peroxidase-conjugated goat anti-mouse IgA or IgG2c (from Southern Biotechnology Associates, Birmingham, AL, USA) was added and incubated for 1 h. The reaction was developed using 2,2′-azino-bis(3-ethylbenzthiazoline-6-sulfonicacid) (ABTS), and the optical density of each well was measured at 490 nm using a Spectra Max 250 Microplate Auto reader (Molecular Devices Corp., Sunnyvale, CA, USA). The results were presented alongside the standard curve, showing data sets that correlated absorbance values to mean concentrations (ng/mL) ± standard deviations, based on averages from three separate experiments.

### 2.7. Determination of Serum Antibody Avidity

The avidity of CT-specific serum IgG and IgG2c antibodies was evaluated using a modified ELISA protocol [37] incorporating antibody standards (for generation of a calibration curve) and ammonium thiocyanate (NH_4_SCN) as a chaotropic agent to disrupt low-affinity antigen–antibody interactions, allowing for the determination of binding strength. A 96-well plate was coated with 10 µg/mL of CT antigen in sodium carbonate buffer, incubated overnight at 4 °C. After washing with PBS-T (0.05% Tween 20 in PBS), the wells were blocked for 1 h with a solution containing 1% BSA and 5% goat serum in PBS-T. Serum samples, diluted 1:20, were added to the wells and incubated at 37 °C for 1 h. The plates were then washed and 2 M NH_4_SCN was added to disrupt low-affinity interactions during a 20 min incubation. The choice of 2 M NH_4_SCN was guided by the results of our optimization studies. We tested NH_4_SCN concentrations from 0.5 to 8 M and 2 M gave the most consistent results. Higher concentrations, such as 4 M, 6 M, or 8 M, showed considerable variability that affected assay reproducibility and accuracy. Following additional washes, HRP-conjugated goat anti-mouse secondary antibody (diluted 1:5000) was added, followed by ABTS substrate, and incubated for 1 h at 37 °C. The reaction was stopped with 1% SDS, and the absorbance was measured at 450 nm with a reference at 650 nm. The relative avidity index was calculated by dividing the mean antibody concentration with NH_4_SCN by the concentration without NH_4_SCN. Avidity was classified as high (above 50%), intermediate (30–49%), or low (below 29%) based on the index values [38].

### 2.8. Assessment of Antigen-Specific Cellular Immune Responses

CT-specific Th1/Th2 cytokine production by SPL and ILN immune T cells was assessed as described previously [20], using the Bio-Plex cytokine assay kit in combination with the Bio-Plex Manager software, SoftMax Pro 7.1 (Bio-Rad, Hercules, CA, USA). Briefly, single cell suspensions were obtained from the SPL and ILN of immunized and control mice 4 weeks after the last immunization using the gentleMACS Dissociator. Immune T cells were purified by positive selection using the Midi magnetic bead-activated cell sorting (MidiMACS) purification system in combination with Pan T cell-specific MACS microbeads (Miltenyi Biotech, Auburn, CA, USA). Equal numbers (2 × 10^5^ cells/well) of purified T cells and mitomycin C-treated splenocytes (APCs) from naïve mice were plated in triplicates in the wells of 96-well tissue culture plates and cultured for 5 days with CT antigen (10 mg/mL). T cells and APCs cultured in the absence of antigen were included as controls. The concentration of the cytokines in each sample was obtained by extrapolation from a standard calibration curve generated simultaneously. The data were calculated as mean values (±S.D.) for triplicate cultures for each experiment.

### 2.9. Statistical Analysis

Statistical analyses were performed with the GraphPad Prism 10 package (GraphPad Software, Inc., La Jolla, CA, USA) on a Mac computer. Statistical differences between groups were evaluated by one-way ANOVA with Tukey’s post-multiple comparison test. Statistical significance was determined at probability (*p*) values ≤ 0.05, 0.01, 0.001, or 0.0001.

## 3. Results

### 3.1. Construction of Plasmid pCT-MECA and VCG Expression of rMECA Protein

A total of 20 predicted T and B cell epitopes from PmpD (10), PmpC (5), PmpG (1), PorB (3), and OmcB (1) made up of 366 amino acids were selected on the basis of high scoring physicochemical properties that included surface accessibility, antigenicity, and hydrophilicity. The 194 amino acid CTA1 was fused to the N-terminal end of MECA via an EAAAK linker. The pCT-MECA vaccine vector was then constructed by inserting the 1980 bp synthesized coding sequence (Figure 1A), which was codon-optimized to enhance expression in *V. cholerae* into the periplasmic targeting vector, pFLAG-CTS, in frame with the Flag Tag sequence under the transcriptional control of the Ptac promoter (Figure 1B). This vector is specifically designed to target expressed proteins in the periplasmic space of Gram-negative bacteria. Sequencing results by GenScript (Piscataway, NJ, USA) confirmed that the inserted gene was in frame with the Flag Tag fusion sequence. The pCT-MECA plasmid was transformed into *V. cholerae* V912 harboring the pDKLO1 lysis plasmid. After gene E-mediated lysis of the cells, the expression of recombinant MECA (~78.88 kDa) was confirmed by Western immunoblotting analysis using anti-Flag monoclonal antibodies (Figure 1C).

### 3.2. IM and IN Vaccination with rVCG-MECA Stimulated Robust Antigen-Specific Antibodies in Serum and Vaginal Secretions

The mice were immunized thrice, at two-week intervals via the IM or IN route, and challenged transcervically, as outlined in the experimental protocol (Figure 2).

**Figure 3:**
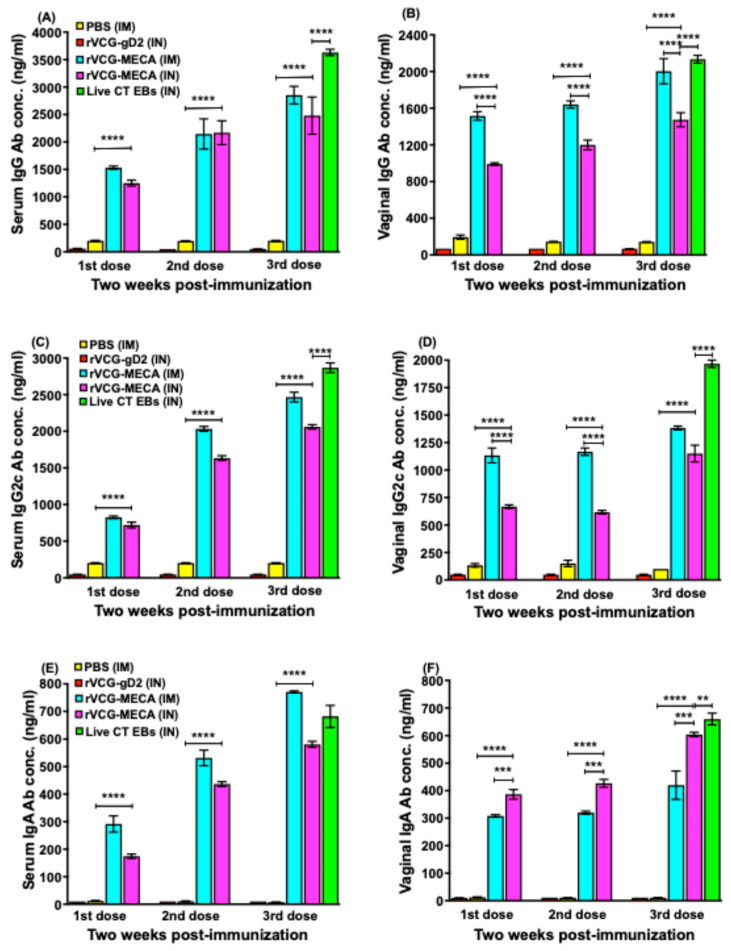
CT-specific systemic and mucosal antibody responses elicited following immunization. Groups of mice were immunized thrice, at two-week intervals via the IM or IN route, as described in the Materials and Methods section. Serum obtained from blood and vaginal lavage samples were obtained two weeks after the first, second, and third immunizations. IgG, IgG2c, and IgA concentrations in serum and vaginal secretions were assessed by a standard antibody ELISA procedure, as described in the Materials and Methods section. The results, from three independent ELISA assays, were simultaneously generated with a standard curve and show data sets corresponding to absorbance values as mean concentrations (ng/mL) ± SD of triplicate cultures for each experiment. The data show the mean antibody concentrations elicited in serum (**A**,**C**,**E**) and vaginal wash (**B**,**D**,**F**) samples. Significant differences between groups were evaluated by one-way ANOVA with Tukey’s post-multiple comparison test at (** *p* < 0.01, *** *p* < 0.001, **** *p* < 0.0001).

CT-specific antibody responses in serum and vaginal wash samples were assessed two weeks after each immunization using a standard ELISA assay. Compared to the PBS and rVCG-gD2 groups, immunization with rVCG-MECA induced significantly higher (*p* < 0.0001) levels of IgG, IgG2c, and IgA antibodies in both systemic and mucosal compartments. These robust antibody responses were observed even after a single dose of the vaccine, regardless of the immunization route (Figure 3). Antibody responses increased with the number of immunization doses with substantial amounts elicited after the third dose. While antibody responses elicited in serum after IM and IN immunization were comparable (Figure 3A,C,E), IM immunization induced significantly higher (*p* < 0.0001) levels of IgG and the IgG2c isotype antibodies in virginal secretions compared to the IN route (Figure 3B,D). In contrast, IgA antibodies were significantly higher (*p* < 0.001) in vaginal secretions following IN immunization (Figure 3F). Antibody responses elicited by immunization with rVCG-MECA were either slightly lower or comparable to levels elicited by IN immunization with live CT EBs (positive control).

**Figure 4:**
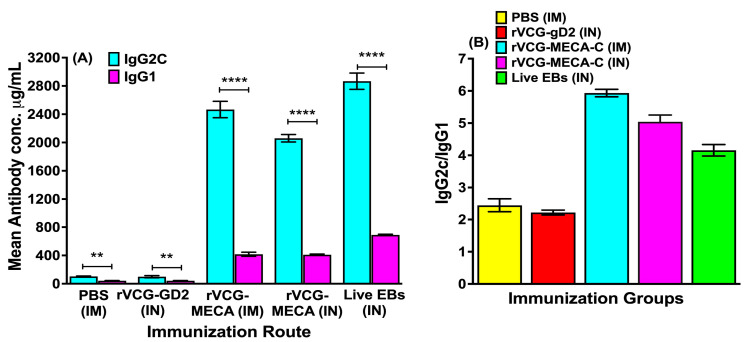
Comparison of the Th1-associated IgG2c and Th2-associated IgG1 antibodies elicited in serum. The mice were immunized thrice, at two-week intervals via the IM or IN route, as described in the Materials and Methods section. Serum separated from blood samples obtained 2 weeks after the last immunization and pooled for each group. CT-specific IgG2a and IgG1 antibody concentrations were measured by a standard antibody ELISA procedure. The results generated simultaneously with antibody standards display the data sets as mean concentrations (ng/mL) + SD of triplicate cultures for each experiment. The data are from one of two independent assays with similar results and show (**A**) the mean IgG2a and IgG1 antibody concentrations and (**B**) the IgG2a/IgG1 ratios. Significant differences between experimental groups were evaluated by one-way ANOVA with Tukey’s post-multiple comparison test at (** *p* < 0.01; **** *p* < 0.0001).

The antigen-specific Th1-associated IgG2c and Th2-associated IgG1 antibodies produced in serum two weeks after the third immunization were also compared. The results showed that IM and IN immunization with rVCG-MECA induced significantly higher (*p* < 0.0001) serum IgG2c compared to IgG1 antibodies (Figure 4A). Further evaluation showed that the IgG2c/IgG1 ratios > 1, irrespective of the immunization route indicating immunization with rVCG-MECA, elicited a Th1-type humoral immune response two weeks post-immunization (Figure 4B).

### 3.3. Vaccine-Induced CT-Specific IgG2c and IgA Antibodies Persisted in Serum and Vaginal Secretions

**Figure 5:**
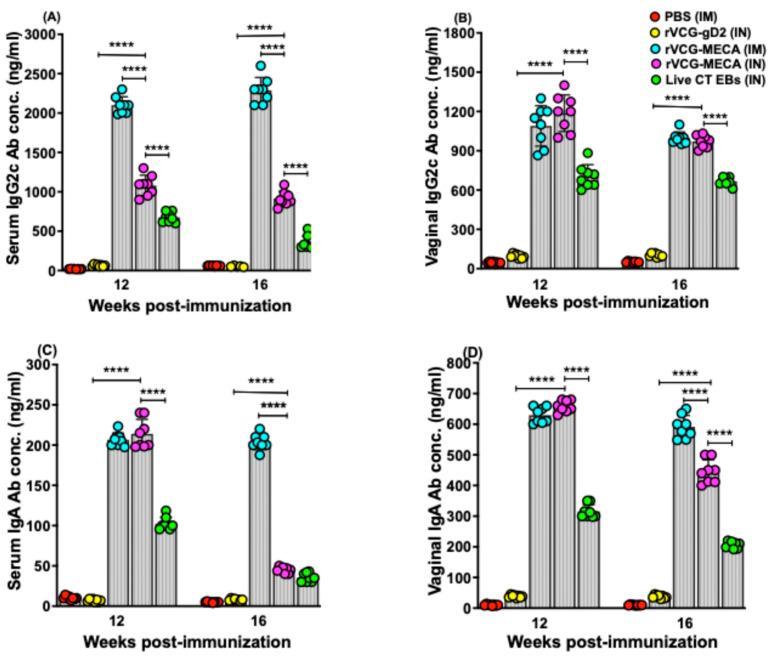
Long-term CT-specific antibody responses elicited after IM and IN vaccination. The mice were immunized as described above. Serum and vaginal secretions were obtained from each mouse per group at 12 and 16 weeks after the last immunization. The antibody levels were measured using a standard ELISA protocol. The results were simultaneously generated with antibody standards and presented the data sets as mean concentrations (ng/mL) + SD of triplicate cultures for each experiment. The data show the individual (distinct colored circles) and mean (represented by each column) IgG2c and IgA antibody concentrations elicited in serum (**A**,**C**) and vaginal wash (**B**,**D**) samples. Significant differences between experimental groups were evaluated by one-way ANOVA with Tukey’s post-multiple comparison test at (**** *p* < 0.0001).

To assess the vaccine’s ability to stimulate prolonged antibody responses, samples collected on days 112 and 140 (12 and 16 weeks post-immunization) were assessed using a standard antibody ELISA assay. The results showed that by week 12 post-immunization, high levels of antigen-specific IgG2c and IgA antibodies were detected in the serum and vaginal secretions of rVCG-MECA-immunized mice, which persisted for up to 16 weeks, irrespective of the route of vaccine delivery (Figure 5A–D). While IgG2c levels in serum were significantly higher (*p* < 0.0001) after IM delivery, comparable levels were elicited in vaginal secretions at both time points (Figure 5A,B). Also, while serum and vaginal IgA levels in both IM- and IN-immunized mice were comparable 12 weeks post-immunization, the levels were significantly lower (*p* > 0.0001) in IN-immunized mice 16 weeks post-immunization (the longest time point evaluated in this study) (Figure 5C,D). The results indicate that the antigen-specific IgG2c and IgA antibody responses elicited in both mucosal and systemic tissues by IM and IN immunization with rVCG-MECA are long lasting.

### 3.4. Avidity of Antigen-Specific Serum IgG and IgG2c Antibodies

**Figure 6:**
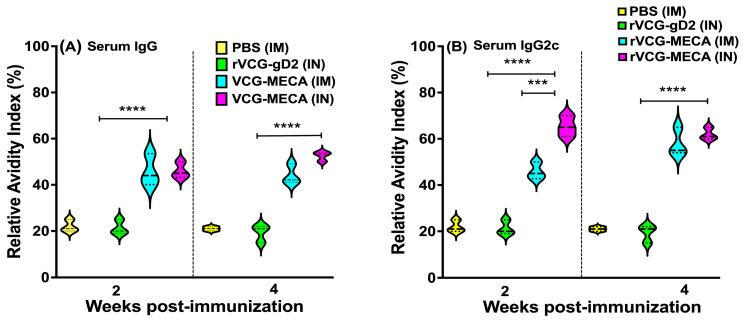
Relative Avidity Index of CT-specific serum IgG and IgG2c antibodies after IM and IN immunization. The avidity of CT-specific IgG and IgG2c antibodies in serum samples from each immunization group was evaluated at 2 and 4 weeks post-immunization using a modified ELISA assay incorporating the chaotropic agent ammonium thiocyanate (NH_4_SCN). The results were generated simultaneously with a standard curve and data sets, corresponding to absorbance values that were calculated as mean concentrations(ng/mL) ± SD of triplicate cultures for each experiment. This experiment was repeated with similar results. The relative avidity index was calculated and displayed as a percentage of the ratio of the antibody concentration of samples treated with NH_4_SCN and the antibody concentration of untreated samples. The data show the percent Relative Avidity Index of serum IgG (**A**) and IgG2c (**B**) antibodies using 2 M NH_4_SCN. Significant differences between experimental groups were compared by one-way ANOVA with Tukey’s post-multiple comparison test at (*** *p* < 0.001, **** *p* < 0.0001).

The functionality of CT-specific IgG and IgG2c antibodies elicited in serum following immunization with rVCG-MECA was evaluated by an ammonium thiocyanate (NH_4_SCN)-based antibody avidity ELISA assay. The data were expressed as relative avidity index, defined as the ratio between the mean antibody concentration in the presence and absence of NH_4_SCN. The data show that the relative avidity index of serum IgG antibodies induced by IM and IN immunization was moderate at 2 weeks post-immunization (Figure 6A). While the avidity of serum IgG antibodies induced by IM immunization remained moderate 4 weeks post-immunization, antibodies with high avidity (>50%) were elicited in IN-immunized mice at this time point (Figure 6A).

The relative avidity index of the Th1-associated serum IgG2c antibodies induced 2 weeks post-immunization was significantly higher (*p* < 0.001) after IN mucosal (65%) compared to IM systemic (46%) immunization (Figure 6B). However, by week 4 post-immunization, the serum IgG2c antibody avidity induced by IM (58%) and IN (62%) immunization was comparable (Figure 6B). In contrast, the relative avidity index of IgG and IgG2c antibodies induced by immunization with PBS (negative control) remained at baseline levels (20%) at both time points (Figure 6A,B). These findings indicate that the Th1-associated IgG2c antibody isotype elicited by rVCG-MECA following both routes of immunization shows higher functional activity compared to total IgG antibodies.

### 3.5. Magnitude of Antigen-Specific IFN-γ Were Induced in Mucosal and Systemic Tissues Following Immunization with rVCG-MECA

**Figure 7:**
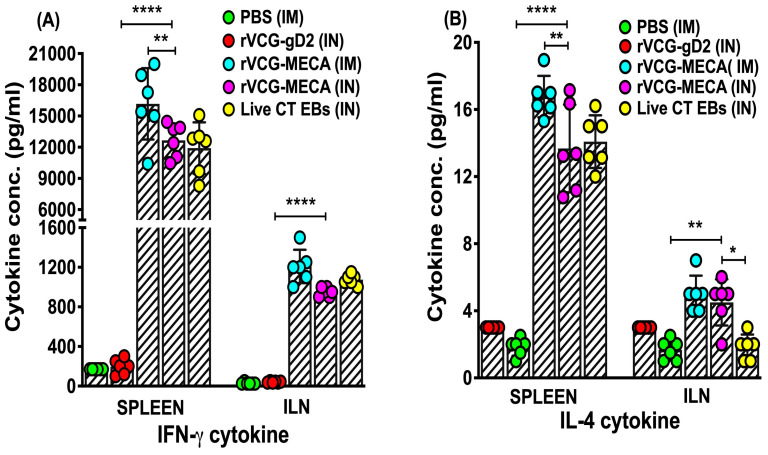
CT-specific mucosal and systemic Th1/Th2 cytokine responses. Immune T cells purified from the spleens and ILNs of immunized mice and controls 4 weeks post-immunization were restimulated in vitro with CT serovar D antigen (UV-irradiated EBs; 10 microgram/mL). The levels of CT-specific IFN-γ (Th1) and IL-4 (Th2) cytokines secreted in the supernatants of culture-stimulated CD4+ T cells were quantified using the Bio-Plex Cytokine Assay kit together with the Bio-Plex Manager software. Cytokine concentrations for each sample were determined by extrapolation from a concurrently generated standard calibration curve. The results are expressed as individual (distinct colored circles) and mean (represented by each column) values (±S.D.) based on quadruplicate cultures for each experiment. The results are from two independent experiments and are shown as mean IFN-g (**A**) and IL-4 (**B**) cytokine concentrations (pg/mL) ± SD. Significant differences between experimental groups were evaluated by one-way ANOVA with Tukey’s post-multiple comparison test at (* *p* < 0.05, ** *p* < 0.01, **** *p* < 0.0001).

Four weeks post-immunization, antigen specific Th1 and Th2 cytokine production was assessed in immune T cells isolated from the spleens and iliac lymph nodes (draining the genital tract) of immunized mice after restimulation with chlamydial antigen in the presence of antigen-presenting cells (APCs). The results show that significantly higher (*p* < 0.0001) levels of the Th1-type cytokine, IFN-γ, were secreted by splenic and ILN immune T cells following both IM and IN immunization compared to T cells from PBS and rVCG-gD2 controls (Figure 7A). While the amount of IFN-γ produced by splenic immune T cells following IM immunization was significantly higher (*p* < 0.01) compared to the IN route, comparable levels were secreted by both splenic and ILN immune T cells following IN immunization with rVCG-MECA and live CT EBs (Figure 7A). Expectedly, immune T cells from the spleens and ILN of PBS- and rVCG-gD2-immunized mice did not secrete substantial levels of IFN-γ. Also, significantly higher (*p* < 0.0001) amounts of IL-4 were secreted by splenic (*p* < 0.0001) and ILN (*p* < 0.01) immune T cells following IM and IN immunization with rVCG-MECA and live CT EBs compared to amounts stimulated by T cells from rVCG-gD2 controls (Figure 7B). While comparable IL-4 levels were produced by ILN immune T cells following IM and IN immunization with rVCG-MECA, IN immunization with rVCG-MECA produced significantly higher (*p* < 0.05) levels than those produced by live CT EBs. However, compared to IFN-γ, considerably lower levels of the Th2 cytokine IL-4 were produced by immune T cells obtained from both spleen and ILN, irrespective of route of vaccine delivery (Figure 7A,B). These results demonstrate that both IM and IN immunizations with rVCG-MECA induce robust Th1-type cytokine responses similar to those induced by immunization with live CT EBs (positive control).

### 3.6. Immune Effectors Stimulated by Immunization with rVCG-MECA Protected Mice Against Transcervical Challenge with Live CT EBs

**Figure 8:**
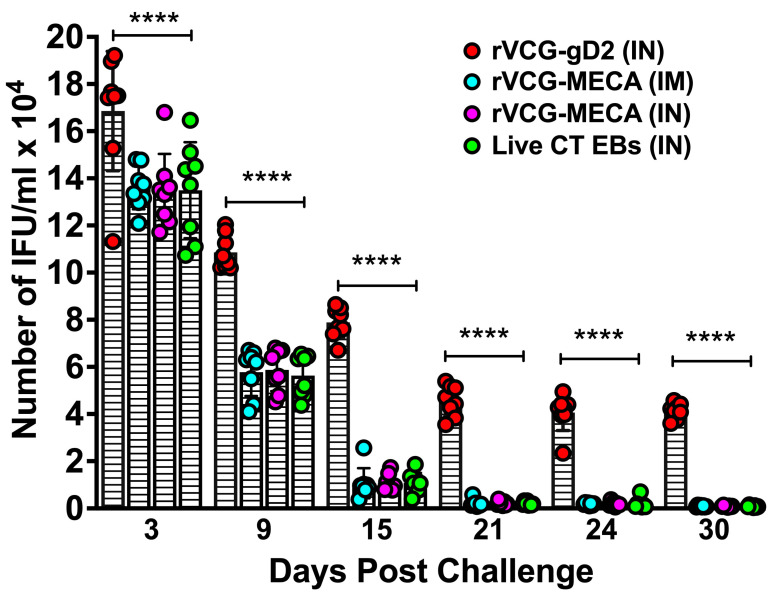
Protection against transcervical challenge with CT serovar D. The mice were immunized IM or IN, as described above, and challenged transcervically with 1 × 10^6^ IFU of live CT 4 weeks after the last immunization. One week before the challenge, the mice were administered Depo Provera to stabilize the estrous cycle and facilitate a productive infection. Infections were monitored by cervicovaginal swabbing of individual animals every three days for 30 days, and *Chlamydia* was isolated from swabs in tissue culture and enumerated. The data show the individual recoverable IFU/mL from each mouse (distinct colored circle) and the mean IFUs for each vaccine (represented by each column). The differences between vaccine groups were compared by two-way ANOVA with Tukey’s post-multiple comparison test at **** *p* < 0.0001.

The protective effectiveness of the rVCG-MECA vaccine was assessed after transcervical CT challenge of immunized mice and controls four weeks after the final immunization. Genital chlamydial loads were evaluated by cervicovaginal swabbing of each mouse every three days, and chlamydial inclusions were enumerated following culture in HeLa cell monolayers. As early as three days post-challenge, there was a significant (*p* < 0.0001) reduction in bacterial load in all immunized mice compared to rVCG-gD2 control mice (Figure 8). A further reduction in chlamydial load was observed in rVCG-MECA vaccine-immunized mice by day 9 post-challenge. Infection loads at this time point were comparable to those in live CT-immunized mice (positive control), irrespective of route of vaccine delivery, but were significantly (*p* < 0.0001) lower than those in rVCG-gD2-immunized mice. All rVCG-MECA vaccine- and CT-immunized mice had started to clear the infection on day 15, and by day 21, all vaccine-immunized groups had completely cleared the infection. rVCG-gD2control mice were still shedding large numbers of CT EBs at this time point.

## 4. Discussion

*V. cholerae* ghosts (VCG) have emerged as an efficient vaccine delivery platform due to their inherent adjuvant properties as they retain all the major immune-stimulating constituents of the bacterial envelope, including lipopolysaccharide, peptidoglycan, toxin co-regulated pili, and certain outer membrane proteins [19], which trigger innate immune responses [39]. The rVCG platform has previously been demonstrated to enhance the protective immunity of vaccine antigens by activating dendritic cells and enhancing antigen presentation [40]. We had also reported that intramuscular systemic and intrarectal mucosal immunization with a rVCG-based multisubunit vaccine based on the polymorphic outer membrane proteins induced robust antigen-specific antibody and cell-mediated immune responses that afforded comparable protection against genital CT infection [21]. This study demonstrated the critical role of the route of vaccine delivery in shaping elicited immune responses and directing them to immune effector sites.

Although VCG-based vaccines are capable of eliciting strong protective immunity without external adjuvants [20,41], our prior research demonstrated that combining a VCG-based subunit *Chlamydia* vaccine with the Fms-like tyrosine kinase 3-ligand (FL), a dendritic cell-targeting adjuvant, significantly enhanced the vaccine’s protective efficacy [21,39], indicating that the use of appropriate adjuvants could further improve the efficacy of VCG-based vaccines. Although intranasal immunization with cholera toxin (Ctx) can target immune effectors to the genital tract, incidence of neurological side effects in experimental mice has prevented its use as a mucosal adjuvant [42]. We previously showed that codelivery of an rVCG vaccine co-expressing the CT major outer membrane protein (MOMP) and CTA2B, the non-toxic derivative of Ctx, significantly enhanced clearance of a *C. muridarum* vaginal infection following intravaginal immunization [43]. Previous studies have demonstrated the effectiveness of CTA1-DD, a non-toxic derivative of Ctx, in stimulating strong CD4+ T-cell and antibody responses, establishing it as a mucosal and systemic adjuvant [24,44]. Also, IN delivery of *C. muridarum* MOMP with CTA1-DD induced neutralizing systemic and mucosal antibodies and reduced genital chlamydial shedding following intravaginal challenge [45]. The immunoenhancing capacity of CTA1 is said to be due to its ability to bind to and directly cause the maturation of immature follicular dendritic cells (FDCs), leading to germinal center (GC) B-cell and follicular helper T-cell (Tfh) development [46]. In this study, we generated a novel subunit vaccine by molecularly fusing CTA1 with a multiepitope CT antigen expressed as a single polycistronic unit (MECA) and expressed in VCG (rVCG-MECA). We evaluated the ability of rVCG-MECA to elicit immune effectors capable of protecting against a transcervical CT infection following the IM and IN routes of vaccine administration. Vaccine evaluation demonstrated that both immunization routes effectively stimulated robust local mucosal and systemic antigen-specific IgG, IgG2c, and IgA antibody responses in serum and vaginal secretions. This is likely due to the combined effect of VCG and CTA1 adjuvant that are known to enhance humoral immune responses in both mucosal and systemic tissues following mucosal and systemic immunization [21,41]. The finding that the IM route elicited significantly higher levels of IgG and IgG2c antibodies in vaginal secretions compared to the IN route indicates that these antibodies likely originate from the systemic circulation. The significantly higher levels of the Th1-associated IgG2c compared to the Th2-associated IgG1 isotype in both mucosal and systemic tissues indicates that immunization with rVCG-MECA skews elicited IgG antibodies toward a Th1 phenotype. This Th1-biased response is crucial for fostering cellular immunity required for the effective clearance of intracellular pathogens like *C. trachomatis* [47,48].

Notably, the IN route more effectively stimulated mucosal immunity following immunization with rVCG-MECA, as demonstrated by elevated IgA levels in vaginal secretions. The elevated IgA concentrations in vaginal washes of IN-immunized mice underscore the ability of this route to elicit mucosal immune responses, which are required for protecting against pathogens such as CT, which establish infections at mucosal surfaces (Nogueira et al., 2017). This result is consistent with other studies where IN immunization was shown to stimulate effective mucosal immunity to subunit antigens [49,50,51]. However, concerns about potential side effects, such as impacts on the central nervous system, have been raised when co-delivered with certain adjuvants [52]. Mucosal routes, such as oral and colonic immunization, have also demonstrated effectiveness in animal models, but they face challenges in delivering subunit vaccines due to the harsh gastrointestinal environment [53,54,55]. Secretory IgA serves as a frontline barrier at mucosal surfaces, preventing pathogen entry, a critical aspect for defense against sexually transmitted infections [52], underscoring the distinct impact of immunization route on humoral mucosal immunity to rVCG-MECA vaccine. While the role of IgA in protection against CT is yet unfolding, previous reports from both mice and human studies indicate that IgA plays an important role in the protection of the genital mucosa [56,57,58]. A recent report showed that local secretory IgA, elicited by oral immunization with either inactivated or live *C. muridarum*, neutralized chlamydial infectivity in the female genital tract, enhancing chlamydial clearance and reducing systemic spread [59]. The robust antibody responses persisted for up to 16 weeks post-immunization, the longest time point assessed, establishing the longevity of mucosal and systemic IgG2c and IgA antibody responses following IM and IN immunization. The ability of this vaccine to provide long-term protection following a genital challenge infection will be evaluated in a future study. The finding that IN-immunized mice produced antibodies with higher binding strength (RAI > 50%) suggests that serum antibodies, especially the IgG2c isotype, induced by mucosal immunization have higher functional capacity, which is crucial for effective pathogen clearance. The Th1-associated IgG2c antibody isotype elicited by rVCG-MECA following both routes of immunization showed higher functional activity compared to total IgG antibodies. While the avidity of IgG antibodies increased at week 4 after IN immunization, the avidity of IgG2c antibodies increased at this time point after IM immunization. The discrepancy in the increase, or lack thereof, in the avidity of total IgG and IgG2c antibodies between the immunization routes is unclear.

The results also showed elevated secretion of the Th1-associated IFN-γ cytokine. Lower levels of the Th2-associated IL-4 cytokine were secreted by splenic and ILN cells following IM and IN immunization, indicating that both the IM and IN vaccine delivery routes effectively stimulated a Th1-type cellular immune response in both mucosal and systemic tissues. The ability of the IM route to induce significantly higher levels of the Th1 cytokine, IFN-γ, compared to the IN route underscores the effectiveness of systemic immunization in stimulating cell-mediated immune responses, which are essential for clearance of intracellular pathogens [48]. Reports from human clinical and animal model studies established the primary role of IFN-γ-secreting CD4+ Th1 cells in protection against CT genital infection [30,47,60,61]. Although CD8+ T cells play a role in protection against the respiratory and genital mucosae [62], its protective role in the female genital tract is incompletely understood. IFN-γ is crucial for macrophage activation, which inhibits the growth of CT enhancing pathogen clearance [63,64]. In addition, some studies have indicated the likely presence of IFN-γ-independent innate mechanisms with the ability to control genital CT infection [65,66]. Although some reports have suggested that a balanced Th1/Th2 response may be beneficial for long-term chlamydial immunity [67], our study demonstrated a predominant Th1 immune response, which, as most studies have indicated, is essential for protection against *Chlamydia* infection.

A number of immunization routes have been employed in the evaluation of the efficacy of subunit chlamydial vaccines. IN immunization with different chlamydial antigens have provided varying degrees of bacterial clearance in the mouse genital tract [68,69]. A previous study demonstrated that mice immunized by a combination of mucosal and systemic routes, involving two mucosal (vaginal + colonic or intranasal + sublingual) and two systemic (IM + SC) routes, afforded the best protection compared those immunized only via the mucosal route [53]. A recent report indicating that a combination of colonic (mucosal) prime followed by IM + SC (systemic) boosts compared to mucosal or systemic only routes provided the best protection confirms this report [49]. Analysis of the protective efficacy afforded by immunization with rVCG-MECA showed that both IM and IN routes provided substantial protection against CT genital infection, as indicated by significant reduction in bacterial load and a shortening of the time to clearance of infection compared to controls. This finding aligns with previous reports demonstrating the ability of VCG-based chlamydial vaccines to induce varying levels of protection following either mucosal or systemic routes of immunization [20,21,41]. These outcomes provide a broader understanding of how the route of administration and the vaccine delivery vehicle impact the protective immunity of CT vaccines. The rVCG-MECA vaccine incorporates immunogenic regions from several key CT antigens, including PmpD, PmpC, PmpG, PorB, and OmcB, which will likely offer broader protection compared to single-antigen vaccines like MOMP-based vaccines. MOMP-based vaccines have been shown to offer serovar-specific protection [18] but are often less effective across multiple serovars [70]. In contrast, our multiepitope approach has the potential to overcome this limitation by targeting multiple regions of the pathogen, offering broader immunity. Other multiepitope vaccines, such as those targeting *C. psittaci*, have also demonstrated the ability to induce robust immune responses and cross-serovar protection [71]. Additionally, a subunit vaccine targeting multiple stages of Mycobacterium tuberculosis showed high protective efficacy in mice, reinforcing the potential of multi-antigen strategies to provide broad protection [71,72]. The inclusion of multiple epitopes in our vaccine construct offers a focused yet comprehensive immune response, avoiding some of the issues associated with whole-antigen vaccines, which may elicit responses to non-protective or pathogenic antigens [51,73].

One of the limitations of this study is the non-inclusion of rVCG-MECA construct that excludes CTA1 to enable a direct evaluation of the effect of CTA1 on vaccine-induced immune responses and protection. Also, the purified rMECA vaccine antigen (without VCG) was not included in this study, which would have enabled evaluation of the effect of the VCG component on vaccine immunity. However, in a preliminary study, we showed that IN immunization with different concentrations of purified rMECA induced substantial serum and vaginal IgG2c and secretory IgA antibodies after two vaccine doses [74]. Studies evaluating the ability of purified rMECA to protect against genital CT infection are ongoing.

## 5. Conclusions

Our findings underscore the importance of considering both the route of immunization and the design of the vaccine when developing strategies to prevent *C. trachomatis* infections. The IM route was highly effective in generating strong systemic Th1 responses, which are crucial for pathogen clearance. The IN route, on the other hand, was highly effective in producing high-avidity IgG2c antibodies and eliciting mucosal immunity through generation of high levels of secretory IgA antibodies, which are critical in defending against CT infection in the vaginal mucosa. Additionally, the ability of rVCG-MECA vaccine to induce robust antibody responses that persisted for up to 112 days post-immunization indicates that it is likely to provide long-lasting protection against CT infection. Therefore, it is a promising candidate for future vaccine development. Future studies will utilize this immunization strategy to evaluate the ability of this multiepitope CT vaccine to protect against heterologous challenges with other chlamydial serovars.

## Data Availability

The data presented in this study are included in the article; further inquiries can be directed to the corresponding author.
